# Perinatal Flavour Learning and Adaptation to Being Weaned: All the Pig Needs Is Smell

**DOI:** 10.1371/journal.pone.0025318

**Published:** 2011-10-19

**Authors:** Marije Oostindjer, J. Elizabeth Bolhuis, Kristina Simon, Henry van den Brand, Bas Kemp

**Affiliations:** Adaptation Physiology Group, Wageningen Institute of Animal Sciences, Wageningen University, Wageningen, The Netherlands; Alexander Flemming Biomedical Sciences Research Center, Greece

## Abstract

Perinatal flavour learning through the maternal diet is known to enhance flavour preference and acceptance of flavoured food in many species, yet still little is known about the mechanism underlying perinatal flavour learning. Previously we found positive effects of perinatal flavour learning on food intake, growth and behaviour of piglets postweaning, but no increased preference for the flavour. This suggests that flavour learning in pigs works through a reduction of weaning stress by the presence of the familiar flavour instead. The aim of this study was to investigate whether perinatal flavour learning reduces stress at weaning, and whether the effect is stronger when the familiar flavour is present in the food. Sows were offered an anethol-flavoured diet (Flavour treatment) or control diet (Control treatment) during late gestation and lactation. Flavour and Control piglets were provided with anethol either in their food (Food treatment) or in the air (Air treatment) after weaning. Preweaning and postweaning treatments did not affect food intake, preference or growth in the first two weeks postweaning but flavour treatment reduced the latency to eat (24 versus 35 hours, P = 0.02) and within-pen variation in growth (SD within-pen: 0.7 versus 1.2 kg, P<0.001). Salivary cortisol levels tended to be lower four and seven hours postweaning for Flavour piglets compared to Control piglets (4 hours: 2.5 versus 3.0 ng/ml, P = 0.05, 7 hours: 3.1 versus 3.4 ng/ml, P = 0.08). Flavour piglets played more and showed less damaging behaviours than Control piglets, indicating that the familiar flavour reduced stress around weaning. Few interaction effects were found between preweaning and postweaning treatment, and no effects of postweaning treatment. We conclude that in the newly weaned pig, perinatal flavour learning results in a reduction of stress when the familiar flavour is present, regardless of providing the flavour in the food or in the air.

## Introduction

Young animals need to make important choices regarding what items to include in their diet around weaning. To make these choices, many animals use information from more experienced conspecifics, because trial-and-error learning is time consuming and potentially lethal [1]. An important source of information is the mother, as young animals generally direct most of their attention to her. Due to their shared genetics, their responses to food types should be very similar and thus the information obtained from her is likely to be relevant [2], [3].

Young animals can start learning which food types are healthy and nutritious from the mother before weaning, and even before birth. Flavours from the maternal diet can reach the foetus before birth through the amniotic fluid, where the flavours are perceived during mouthing movements or ingestion of the amniotic fluid [4], [5]. Flavours that cross the placental barrier may also reach the foetus via the foetal blood stream, where they can be perceived in the nasal capillaries [6]. Studies on prenatal flavour learning have shown that the offspring of several species, such as humans, rats, dogs, rabbits, cats, sheep and even chickens and frogs, show a preference or reduced aversion to flavours to which they have been exposed before birth or hatching [6], [7], [8], [9], [10], [11], [12], [13], [14], [15], [16], [17], [18]. Additionally, flavours may reach young mammals after birth through the mother's milk. This continued exposure may strengthen the preference for these flavours as seen in humans, rats, dogs and rabbits [19], [20], [21], [22], [23], [24]. Thus, at weaning, young animals will more readily accept foods containing flavours to which they have been exposed through the maternal diet. This is due to an increases preference for these flavours [25], [26].

In our earlier work we investigated whether perinatal flavour learning would increase preference and stimulate intake of flavoured foods in piglets [27], [28]. We chose the pig as a model as perinatal flavour learning might potentially be used to improve piglet health and welfare in the immediate postweaning period. Weaning in husbandry occurs abruptly and at an earlier age than in the wild. As a result, many piglets are not yet adapted to eating solid food at weaning, making weaning a stressful event for piglets under husbandry conditions [29]. We found that piglets exposed to anise flavour (anethol) through the maternal diet, in particular prenatally, recognized anise flavour during lactation, but did not show a clear preference for it [27]. Moreover, in mildly challenging situations prenatally exposed piglets showed less signs of stress in the presence of the flavour than non-exposed piglets. In the early postweaning period perinatal flavour learning positively affected food intake and growth of piglets, but exposed piglets again showed no preference for the anise, and typically chose control food over the flavoured food that was provided [28]. It is possible that piglets were initially attracted to the flavoured food but quickly generalized to the control food [30], [31], resulting in an increased overall food intake. On the other hand, the mere presence of the familiar flavour in the postweaning environment may have reduced stress [27], [28], [32]. Odours that are familiar to the piglet, for example through association with the mother or the safe and familiar home cage, can reduce stress-related behaviours and neophobia as demonstrated in humans and chickens and our previous study on pigs [27], [33], [34], [35]. These odours may have been responsible for reduced weaning stress, which would account for the positive effects of perinatal flavour learning in newly weaned piglets [28].

The aim of this study was to gain more insight in the mechanism of perinatal flavour learning by investigating whether perinatal flavour learning and consequent re-exposure to the flavour can reduce stress of piglets after weaning. We investigated if it is necessary to have the familiar flavour in the postweaning food, or if the same effects on stress and adaptation to weaning can be obtained by having the familiar flavour present in the air.

## Results

### Cortisol levels on day of weaning

**Figure 1:**
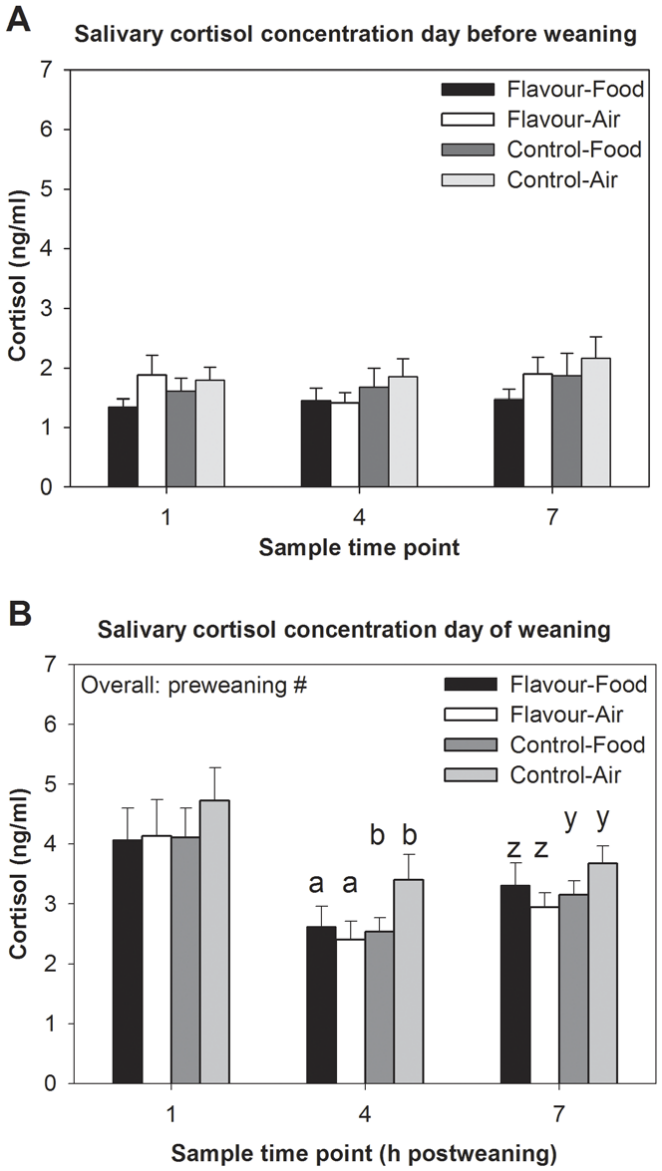
Salivary cortisol levels. Salivary cortisol levels on the day of before (**A**) and the day of weaning (**B**), sampled at the same time points on the day, for piglets exposed to Flavour through the maternal diet or Control piglets, housed in postweaning pens containing the flavour in the Food or in the Air. #: P<0.1. Different superscripts indicate significantly different values (a/b) and values that tend to be different (z/y).

Preweaning treatment tended to affect cortisol levels over the three time points measured postweaning, with piglets in the Flavour treatment showing lower levels than Control piglets (F_(1,43)_ = 3.53, P = 0.07, Figure 1). Flavour-exposed piglets showed lower cortisol levels four hours after weaning (F_(1,41)_ = 4.17, P = 0.05) and tended to have lower cortisol levels seven hours after weaning (F_(1,42)_ = 3.19, P = 0.08). Cortisol levels over the three time points measured postweaning were unaffected by postweaning treatment (P = 0.57), or by an interaction between pre- and postweaning treatment (P = 0.21).

When looking at the differences in cortisol levels between the day before weaning (basal samples) and the day of weaning, the Control-Air piglets showed the highest increase in cortisol concentration at the three time points (Control-Air: 2.29±0.4 ng/ml, Flavour-Food: 2.12±0.4 ng/ml, Flavour-Air: 1.66±0.4 ng/ml, Control-Food: 1.58±0.5 ng/ml preweaning×postweaning interaction, F_(1,43)_ = 4.52, P = 0.04). Control-Air piglets showed higher cortisol levels than Control-Food and Flavour-Air piglets four hours after weaning (Flavour-Food: 1.5±0.3, Flavour-Air: 1.1±0.3, Control-Food: 0.9±0.3, Control-Air: 2.0±0.4, preweaning×postweaning, F_(1,41)_ = 4.28, P = 0.04). A preweaning×postweaning interaction was found seven hours after weaning as well (F_(1,42)_ = 3.12, P = 0.09). Preweaning treatment (P = 0.63) and postweaning treatment (P = 0.62) did not affect the increase of cortisol concentrations.

### Latency to eat, food intake and growth

**Figure 2:**
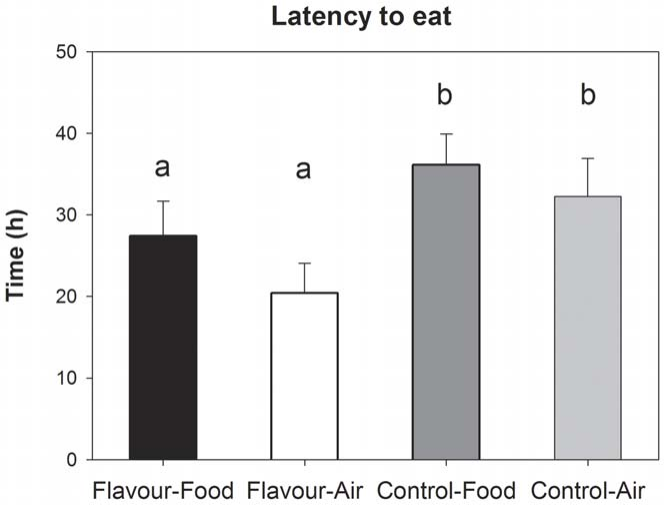
Latency to eat. Latency to eat for piglets exposed to Flavour through the maternal diet or Control piglets, housed in postweaning pens containing the flavour in the Food or in the Air. Different superscripts indicate significantly different values (a/b).

Latency to eat is presented in Figure 2, and other measures of food intake and growth are presented in Table 1. Food intake and body weight were unaffected by preweaning and postweaning treatments on any particular day and only presented over the total postweaning period.

**Table 1:** Growth and food intake after weaning^1^.

	Treatment				P-values		
	Flavour-Food	Flavour-Air	Control-Food	Control-Air	Pre	Post	Prexpost
Total food intake postweaning (kg)	18.8±1.3	18.9±0.9	18.3±1.4	18.7±1.0	0.65	0.73	0.86
Total anise intake postweaning (kg)	4.0±1.3	-	3.7±1.3	-	0.91	-	-
Weaning weight (kg)	8.41±0.25	8.54±0.23	8.46±0.25	8.58±0.24	0.84	0.62	0.99
Total growth postweaning (kg)	3.80±0.27	3.95±0.21	4.16±0.29	3.86±0.27	0.33	0.59	0.11
Variation growth within pen (kg)	0.72±0.08^a^	0.66±0.11^a^	1.12±0.12^b^	1.04±0.09^b^	<0.001	0.42	0.91
Pigs losing weight in the first 3 days (%)	16.7±6.4	6.2±3.3	25.0±10.2	18.7±8.2	0.13	0.40	0.90

1 Different superscripts indicate significantly different values.

Piglets that were exposed to anise flavour before weaning showed a shorter latency to their first meal than control piglets (preweaning treatment: F_(1,43)_ = 6.34, P = 0.02). Postweaning treatment (P = 0.18) and its interaction with preweaning treatment (P = 0.70) did not affect the latency to eat. Total food intake in the first two weeks postweaning was unaffected by preweaning treatment (P = 0.65), postweaning treatment (P = 0.73) and by the pre- and postweaning treatment interaction (P = 0.86). The amount of anise food consumed by the piglets that had anise flavour in the postweaning food was unaffected by preweaning treatment (P = 0.91). Total growth was unaffected by preweaning treatment (P = 0.33), postweaning treatment (P = 0.59) or the treatment interaction (P = 0.11), but the variation in postweaning growth within pens was smaller for piglets exposed to flavour before weaning (F_(1,43)_ = 19.2, P<0.001). Within-pen variation in growth was unaffected by postweaning treatment (P = 0.42) and the interaction between pre- and postweaning treatment (P = 0.91). The percentage of piglets within pen that lost weight in the first three days was not affected by preweaning treatment (P = 0.13), postweaning treatment (P = 0.40) or the treatment interaction (P = 0.90). Numerically, however, fewer piglets in the preweaning Flavour group lost weight in the first three days postweaning, particularly the piglets in the Flavour-Air group.

### Behaviour

**Figure 3:**
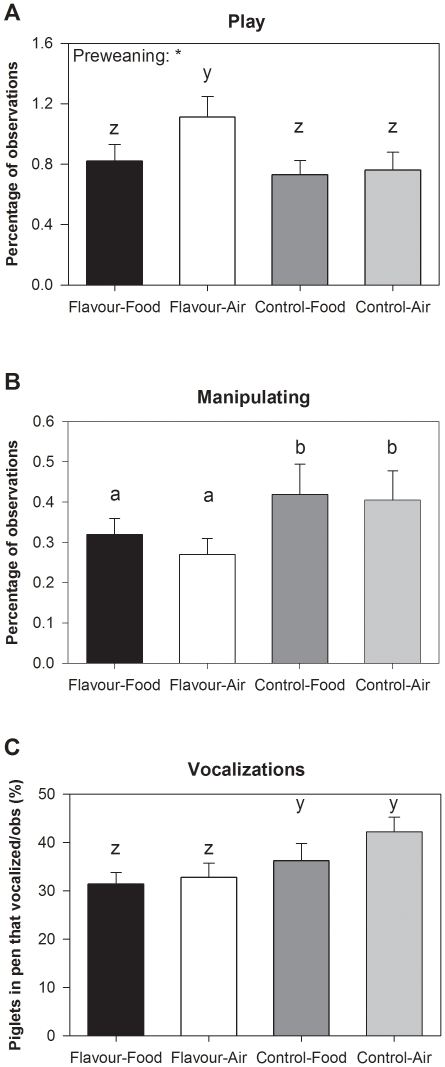
Behaviour after weaning. Play behaviour (**A**), manipulative behaviour (**B**) and percentage of piglets per pen that vocalized on day 1 after weaning (**C**). Piglets were exposed to Flavour through the maternal diet or were Control piglets, housed in postweaning pens containing the flavour in the Food or in the Air. Please note the differences in scale. *: P<0.05. Different superscripts indicate significantly different values (a/b) and values that tend to be different (z/y).

Piglets that were exposed to flavour preweaning showed more play behaviour in the first two weeks postweaning (F_(1,43)_ = 7.60, P = 0.009, Figure 3A). Play behaviour tended to be higher for Flavour-Air piglets than for piglets from other treatments (preweaning×postweaning interaction, F_(1,43)_ = 3.17, P = 0.08, Figure 3A) and was unaffected by postweaning treatment (P = 0.16). Piglets that were exposed to flavour before weaning tended to manipulate pen mates less than control piglets (F_(1,43)_ = 3.22, P = 0.08, Figure 3B). Manipulative behaviour was unaffected by postweaning treatment (P = 0.51) and its interaction with preweaning treatment (P = 0.54). The percentage of pigs that vocalized per pen tended to be lower for the Flavour piglets on day 1 after weaning than for Control piglets (F_(1,35)_ = 3.93, P = 0.06, Figure 3C). Vocalizations were unaffected by postweaning treatment (P = 0.45) and its interaction with preweaning treatment (P = 0.47).

## Discussion

Although many studies have examined perinatal flavour learning in a wide range of species in the last four decades, there is still little known about the mechanisms underlying flavour learning and how perinatal flavour learning results in an increased acceptance of food items [6], [7], [8], [9], [10], [11], [12], [13], [14], [15], [16], [17], [18], [19], [20], [21], [22], [23], [24], [25], [26]. Our earlier work [27], [28] suggests that perinatal flavour learning does not necessarily result in an increased preference for flavoured food items in pigs when the pigs are tested in a challenging environment. Perinatal flavour learning did, however, positively affect piglet growth, food intake and behaviour during re-exposure in such a challenging environment. This suggests that perinatal flavour learning may result in lower stress levels during re-exposure in a stressful environment, which in turn can facilitate the acceptance of flavoured food items, irrespective of preference.

This study indicates that perinatal flavour learning followed by re-exposure to the familiar flavour at weaning indeed may reduce stress in piglets. Flavour-exposed piglets tended to show a faster decrease in salivary cortisol levels after weaning, indicating that the initial stress response was similar for all piglets but that the familiar flavour resulted in a faster recovery. The seemingly reduced stress levels were also reflected in the piglets' behaviour, with a tendency for less vocalizations on the day after weaning and more play behaviour after weaning. Low levels of vocalizations and high levels of play after weaning indicate lower stress levels induced by weaning-related stressors such as maternal separation, relocation and general frustration [32], [36], [37], [38]. There were also effects on the longer-term on variation in growth and behaviour that indicate that stress was reduced and piglets were better able to adapt to the postweaning situation [32], [36], [37], [38], [39], [40]. Animals are generally more reluctant to eat and try novel foods in unfamiliar environments [41] and the shorter latency to ingest solid food of flavour-exposed piglets indicates that the familiar flavour either made the food less novel or the pen less unfamiliar. Time to eat the novel food, as well as cortisol, behaviour and growth, did not differ between piglets that received the flavour in the food or in the air. It seems thus not necessary to provide the flavour in the food, which suggests that it is particularly the odour component of the familiar flavour that reduces stress.

Odours that are familiar and associated with a positive experience or environment can result in a strong positive memory recall and consequently affect mood, referred to as the Proust phenomenon [42], [43], [44], [45]. The association of the odour with a positive context may be strongest when the context is related to the mother [46], [47], though it is not necessary to learn these associations in this particular positive context to affect emotionality and mood [42], [48]. It seems, however, that for hedonic or aversive valence of flavours, it is necessary to make the association with the odour with either a positive or negative experience or environment [49]. The amygdala plays an important role in making both positive and negative associations, as well as in creating the emotions during re-exposure to the odour and consequent memory evocation [50], [51], [52]. Although most studies that looked at how familiar odours or flavours affect emotionality, memory evocation and mood have been performed in humans, there is also evidence from work on chickens and our earlier work in pigs that familiar flavours can also reduce stress and possibly influence emotionality in animals [27], [35], [53]. In the current study, flavours in the amniotic, which is a very attractive and positive substrate for young animals [54], [55], [56], [57], may have resulted in an association of the flavour with a very positive context: the mother. Whether flavours in the prenatal or postnatal environment are more important for the learning of the association remains unclear from this study, but our previous work in pigs showed practically no effects of postnatal exposure only (from day 6 onwards) [27], [28]. This suggests that the association is made prenatally or in the very early postnatal period, although continuity of flavour exposure might be important to strengthen the association [20], [58]. The association of the flavour with a familiar environment may have positively affected emotions of piglets at weaning when they were re-exposed to the flavour in a stressful situation, thereby reducing stress levels and possibly enhancing ‘mood’. Higher levels of play behaviour by the piglets that were previously exposed to the flavour provide some support for the idea that their perception of the postweaning period was less negative than that of control animals. The more familiar odour of the pen, due to the presence of the flavour, may also have made the postweaning pen a less novel experience, which may have increased adaptation to weaning and reduced the negative effects that are generally induced by weaning [29].

The results also suggest that the positive valence of the flavour, or the odour component of the flavour, may not be generalized to the taste component of the flavour in newly weaned piglets. Flavour-exposed piglets did not show a preference for the anise food, which is in contrast to most studies on perinatal flavour learning that do find a specific preference for and acceptance of foods that are flavoured with the familiar flavour [7], [10], [11], [12], [13], [15], [16], [24], [59]. The strong preference of the control food over the anise food by piglets in this study suggests that the palatability of the food was reduced by addition of the flavour, and the use of a highly preferred flavour would likely have positive effects on piglet behaviour when provided in the food. Perinatal flavour learning, however, can reduce the reluctance to accept foods with low palatability [26], [60], [61], [62], but this was not observed in the current study, possibly due to the two-choice feeding protocol used. Despite this, it still seems that the effects of perinatal flavour learning are different in newly weaned piglets than in other species tested. In our view, the positive association with the mother may have overruled any other effects on preference due to the re-exposure to the flavour in a very stressful situation (weaning).

In conclusion, it seems that perinatal flavour learning can result in a reduction of stress when the familiar flavour is present in a challenging situation. Also, the smell of the familiar flavour alone seems enough to obtain the stress-reducing effects.

## Materials and Methods

### Ethics statement

This study was carried out in strict accordance with the recommendations in the European Guidelines for accommodation and care of animals. The protocol was approved by the Institutional Animal Care and Use Committee of the University of Wageningen (Protocol Number: 2010014c).

### General setup

The experiment was set up in a 2×2 factorial design including postweaning pen treatments, with preweaning treatment (Flavour or Control) and postweaning treatment (flavour in Food or flavour in Air) as factors. The experiment was conducted in two successive batches. Animals could be individually recognized through-out the experiment.

### Animals and housing preweaning

A total of 24 multiparous PIC Camborough sows (commercial synthetic sow line, includes Landrace and Large White breeds) and their offspring (Tempo (commercial synthetic boar line with Great York genetic background)×PIC Camborough) were used. Sows were assigned to the Flavour (n = 12) or Control (n = 12) treatment group and received anise flavoured or control food, respectively, between days 98 and 115 of gestation (sows generally give birth on day 115 of gestation), and from day 2 after giving birth until weaning the piglets on day 25.

Sows were individually housed in four different rooms (2 flavour, 2 control) per batch from day 95 of gestation onwards, in farrowing pens of 3.5×2.2 m. Sows were placed in a farrowing crate (2.2×0.6 m) within the pen from day 115 of gestation until 3 days after birth of the piglets. All pens had a small layer of wood shavings and were provided with approximately 100 grams of straw daily. Litters of piglets were standardized to 10–12 piglets, when necessary, by cross-fostering within treatment groups before day 3 after birth. Both males and females were used. A personality test (backtest) was performed on day 10 to classify piglets as either high resisters (more active coping response to stress and novelty) or low resisters (more passive coping response to stress and novelty), [63], [64], in order to make a balanced assignment of piglets to pens postweaning (see below). No food was provided to the piglets during the lactation period, but water from drinking nipples was available to all piglets. Lights were on between 7:00 and 19:00.

### Flavour exposure protocol

Anise was chosen as the experimental flavour and provided to sows and piglets in the same dosage as reported previously [27], [28]. Sows in the flavour group received a daily dose of 350 mg trans-anethol (99%, Sigma-Aldrich), which is the molecule responsible for the anisic flavour, given in two daily portions of 175 mg. The portions of 175 mg anethol were dissolved into 20 ml soy oil and kept in 20 ml syringes in the dark to prevent the anethol from disintegrating. Control sows received two daily portions of 20 ml soy oil. Control syringes were kept in a separate location in the experimental building to prevent contamination.

The anethol solution (or soy oil) was sprayed on top of a portion of 300 grams of food (standard commercial unflavoured sow diets, same food for Control and Flavour sows), which was between 4 and 10% of the total daily food intake of sows, dependent upon the gestational and lactational stage. Additional food was not given until the sow finished the small portion of food. All sows in the flavour group consumed the anise and oil treated food. The protocol to prevent exposure of control sows and piglets to the anethol included feeding control sows before flavour sows, wearing gloves, transporting anethol-contaminated objects in plastic bags, and changing clothes after entering the flavour rooms.

### Animals and housing postweaning

Piglets were weaned at 25±2 days and housed in pens of 2.1 m×2.8 m, with in total four unfamiliar piglets per pen from the same preweaning treatment. Only eight pigs per litter were used, of which four piglets were assigned to the Food treatment (flavour in food), and four to the Air treatment (flavour in air), resulting in 12 Flavour-Air pens, 12 Flavour-Food pens, 12 Control-Air pens and 12 Control-Food pens. Each pen contained two males and two females, with one of each sex being a high resister and one being a low resister, as defined by the backtest. Weaning weights of piglets were balanced between postweaning treatments (see Table 1 for weaning weights).

Each pen had two feeders, one on each side of the pen. Each pen also had an air permeable container that was placed in the back of the pen and could be touched but its contents could not be accessed by the piglets. The Food treatment pens had one feeder with control food (standard weaner diet, SpeenSelect, Rijnvallei, different composition and base flavour than sow's diet) and one feeder with control food with 150 ppm of powdered anethol added. The permeable container in the Food treatment pens contained 1 kg of control food. The feeders in the Air group both contained control food, while the permeable container in these pens contained 1 kg of 150 ppm anethol-flavoured food, thus the flavour was only present in the air in these pens. For both postweaning treatment groups the powdered anethol (Lucta S.A., Spain) was mixed into the food for the feeders and containers as designated above at the moment of provisioning. Food in the feeders and containers was replaced on days 2, 5, 8 and 11 postweaning. Each pen was provided with a small layer of wood shavings as bedding and approximately 50 g of straw per day. Lights were on at 7:00 and off at 19:00. Water was available from two drink nipples in each pen.

### Postweaning measurements

Piglets were weighed at day 0, 3, 7, 11 and 14 postweaning. Food intake was measured by weighing back the feeders at 5 h, 24 h, days 2, 3, 5, 8, 11 and 14 postweaning. Latency to eat was determined by video observations, in which the time between weaning and the first time the piglet spent more than three seconds with its head deep in the feeder was calculated for each individual piglet. If a piglet had not eaten after the third day, the maximum score was given for this piglet, which was the time from weaning until midnight on the third day (n = 10, maximum scores between 61 and 63 hours).

Saliva samples for cortisol measurement were collected on the day of weaning at 1, 4 and 7 h postweaning from the two males piglets from each pen. The males were sampled as female piglets tend to handle the stress associated with weaning better than males [65]. Basal samples of these piglets were collected on the day before weaning at the same time points on the day. Piglets were presented with three cotton swabs on which they readily chewed after they had been habituated to the swabs three and two days before weaning. Swabs were collected into Salivettes® and stored on ice until all piglets for that time point were sampled. Salivettes were then centrifuged at 3000 RPM (870 RCF) for 10 minutes and stored at −20°C until analysis. Salivary cortisol was determined using a solid-phase radioimmunoassay kit (Coat-a-Count Cortisol TKCO, Diagnostic Products Corporation, Apeldoorn, The Netherlands) modified for pig salivary cortisol as described in a previous study [66].

Behaviour of piglets in their pen was scored on day 1, 5, 9 and 13 after weaning using 2-min instantaneous scan sampling for 6 h per day, resulting in 180 observations (scans) per piglet per day. Data were collected using the Psion Workabout MX with the Observer 5.0 (Noldus Information Technology B.V., Wageningen, The Netherlands) installed on it. Behaviours that indicate whether a piglet adapted to weaning well are play behaviour (running around the pen, pivoting, rolling, sliding, gambolling or substrate play) and manipulating pen mates (nibbling, sucking or chewing body parts of pen mates). Play was expected to be lower in piglets that experienced the weaning process as very stressful [32], [37], and manipulative behaviour was expected to be higher in piglets that experienced the weaning process as very stressful [32], [37]. The percentage of piglets per pen that vocalized during behaviour sampling for that specific pen was also determined every two minutes on day 1 after weaning for 6 h, as high vocalization rates postweaning indicate stress or frustration [27], [36], [38].

### Data analysis

All data were analysed using mixed linear models in SAS (SAS 9.1, SAS Institute Inc.). Behaviours (expressed as proportions of time) were arcsine-square root transformed when the residuals were not normally distributed. Effects of pre- and postweaning treatments on cortisol levels on the day of weaning and on the difference between basal levels and levels on the day of weaning were analysed with a repeated model. This model included preweaning treatment (Control or Flavour), postweaning treatment (Food or Air), day and their interaction, as well as batch as main factors, and postweaning pen and piglet as random factors (testing against pen). Growth, food intake, latency to eat, home pen behaviour, vocalizations and cortisol levels by time point were analysed with a model including pre- and postweaning treatments, their interactions and batch as main factors, and pen within treatment as random effect. Within-pen variation of growth (standard deviations) was also analysed with this model. Post-hoc pairwise comparisons were made using the least-square means, corrected for multiple comparisons with a Tukey adjustment. Data are presented as (untransformed) mean ± SEM based on pen averages.
